# Differential effects of novel kappa opioid receptor antagonists on dopamine neurons using acute brain slice electrophysiology

**DOI:** 10.1371/journal.pone.0232864

**Published:** 2020-12-29

**Authors:** Elyssa B. Margolis, Tanya L. Wallace, Lori Jean Van Orden, William J. Martin

**Affiliations:** 1 Department of Neurology, UCSF Weill Institute for Neurosciences, University of California, San Francisco, San Francisco, CA, United States of America; 2 BlackThorn Therapeutics, San Francisco, CA, United States of America; University of Pittsburgh, UNITED STATES

## Abstract

Activation of the kappa opioid receptor (KOR) contributes to the aversive properties of stress, and modulates key neuronal circuits underlying many neurobehavioral disorders. KOR agonists directly inhibit ventral tegmental area (VTA) dopaminergic neurons, contributing to aversive responses (Margolis et al. 2003, 2006); therefore, selective KOR antagonists represent a novel therapeutic approach to restore circuit function. We used whole cell electrophysiology in acute rat midbrain slices to evaluate pharmacological properties of four novel KOR antagonists: BTRX-335140, BTRX-395750, PF-04455242, and JNJ-67953964. Each compound concentration-dependently reduced the outward current induced by the KOR selective agonist U-69,593. BTRX-335140 and BTRX-395750 fully blocked U-69,593 currents (IC_50_ = 1.2 ± 0.9 and 1.2 ± 1.3 nM, respectively). JNJ-67953964 showed an IC_50_ of 3.0 ± 4.6 nM. PF-04455242 exhibited partial antagonist activity asymptoting at 55% blockade (IC_50_ = 6.7 ± 15.1 nM). In 3/8 of neurons, 1 μM PF-04455242 generated an outward current independent of KOR activation. BTRX-335140 (10 nM) did not affect responses to saturating concentrations of the mu opioid receptor (MOR) agonist DAMGO or the delta opioid receptor (DOR) agonist DPDPE, while JNJ-67953964 (10 nM) partially blocked DAMGO and DPDPE responses. Importantly, BTRX-335140 (10 nM) rapidly washed out with complete recovery of U-69,593 responses within 10 min. Collectively, we show electrophysiological evidence of key differences amongst KOR antagonists that could impact their therapeutic potential and have not been observed using recombinant systems. The results of this study demonstrate the value of characterizing compounds in native neuronal tissue and within circuits implicated in the neurobehavioral disorders of interest.

## Introduction

One of the major challenges in drug development is predicting whole animal responses based on pharmacological characterization in heterologous systems. Recent biological reports indicate that the effect of drugs on G protein coupled receptor function *in situ* in brain tissue is not reliably predicted from results in expression systems [1–6]. Therefore pharmacological characterizations made in brain tissue likely relate better to behavioral outcomes than those made in cell-based expression assays.

Interest in the kappa opioid receptor (KOR) as a target for therapeutic development has been growing consistently as clinical and preclinical studies have identified its role in aversive behavioral states. KOR agonists produce profound adverse effects in humans, specifically fatigue, sedation, confusion, impaired concentration, and anxiety. Furthermore at higher concentrations visual and auditory hallucinations and feelings of depersonalization have been reported [7, 8]. Homologous effects have been described in animal models (reviewed in [9]). Finally, blockade or genetic deletion of the KOR significantly reduces aversive responses to stress [10–12], drug withdrawal [13–15], and pain [16], and has antidepressant-like effects [17] in preclinical models, suggesting that KOR selective antagonists could be useful therapeutic agents.

Historically, the known synthetic KOR antagonists, including the most widely used KOR antagonist for laboratory research norbinaltorphimine (norBNI), have properties limiting their clinical potential, including long lasting blockade of KOR agonist activity [18, 19]. These long lasting effects have been alternatively attributed to prolonged retention time in the brain [20] or a signaling process involving the activation of the c-Jun N-terminal kinase (JNK) pathway [21, 22]. In addition, some possess poor selectivity for KOR over other opioid receptors and have other off-target effects [23, 24]. Recently, new compounds have been synthesized to overcome these limitations [25]. In particular, BTRX-335140 (1-(6-ethyl-8-fluoro-4-methyl-3-(3-methyl-1,2,4-oxadiazol-5-yl)quinolin-2-yl)-*N*-(tetrahydro-2H-pyran-4-yl)piperidin-4 amine) has been reported to have a medication-like duration of action [26] and is currently in clinical trials. PF-04455242 (2-methyl-N-((2'-(pyrrolidin-1-ylsulfonyl)biphenyl-4-yl)methyl)propan-1-amine; Pfizer) [27] and JNJ-67953964 (formerly LY2456302 / CERC-501) (S)-3-fluoro-4-(4-((2-(3,5-dimethylphenyl)pyrrolidin-1-yl)methyl)phenoxy)benzamide) also have been in clinical development as selective KOR antagonists.

In rats, the model species used here, KOR activation in the VTA is aversive and directly inhibits the activity of dopamine neurons [28, 29]. Several brain regions that innervate the VTA express mRNA for the endogenous KOR ligand, dynorphin, including ventral striatum, amygdala, and lateral hypothalamus [30–32]. One hypothesis is that a major contributor to maladaptive aversiveness is dynorphin release from one or more of these inputs, inhibiting dopamine neuron activity that KOR antagonist treatment would reverse, generating relief [33–35]. Therefore, here we investigated properties of 4 synthetic KOR antagonists in VTA dopamine neurons, a locus implicated in some of the proposed clinical indications for KOR antagonist treatments [9]. We used an acute midbrain slice and whole cell electrophysiology preparation to evaluate the potency, selectivity, and reversibility of a selection of recently developed KOR antagonists to gain a better understanding of their pharmacologies in brain tissue.

## Materials and methods

All animal protocols were conducted in strict accordance with the recommendations of the National Institutes Health (NIH) in the Guide for the Care and Use of Laboratory Animals. Research protocols were approved by the Institutional Animal Care and Use Committee (University of California at San Francisco, CA), approval ID AN169369-3B. Sacrifice via decapitation was performed after deeply anesthetizing the rats with isoflurane to minimize discomfort.

### Tracer injections

Male Sprague Dawley rats (27–29 d, 5 rats) were anesthetized with isoflurane and secured in a skull stereotax. A glass pipette tip attached to a Nanoject II (Drummond Scientific, Inc.) was stereotaxically placed in the medial prefrontal cortex (mPFC) (from bregma (in mm): anteroposterior (AP), +2.6; mediolateral (ML), ±0.8; ventral (V), −4.0 from skull surface). Neuro-DiI (7% in ethanol; Biotium) was slowly injected, 200 nL per side. All injection sites were histologically examined and only data collected from rats with on target injections were included in the analysis.

### Slice preparation and electrophysiology

Recordings in retrogradely labeled neurons were made 7–8 d after surgery (~P35). For all other experiments, male SD rats were used, P23 –adult, as indicated in Table 1. Rats were deeply anesthetized with isoflurane and then sacrificed by decapitation with a guillotine. Horizontal brain slices (150 μm thick) were prepared using a vibratome (Leica Instruments). Slices were prepared in ice-cold aCSF (in mM: 119 NaCl, 2.5 KCl, 1.3 MgSO_4_, 1.0 NaH_2_PO_4_, 2.5 CaCl_2_, 26.2 NaHCO_3_, and 11 glucose saturated with 95% O2−5% CO_2_) and allowed to recover at 33°C for at least 1 h. Slices were visualized under a Zeiss AxioExaminer.D1 with differential interference contrast, Dodt, and near infrared optics, and epifluorescent illumination to visualize DiI-labeled neurons. Whole cell recordings were made at 33°C using 3–5 MΩ pipettes containing (in mM): 123 K-gluconate, 10 HEPES, 0.2 EGTA, 8 NaCl, 2 MgATP, 0.3 Na_3_GTP, and 0.1% biocytin (pH 7.2, osmolarity adjusted to 275). Liquid junction potentials were not corrected during recordings.

**Table 1:** Rat ages for recordings.

	Dose Response (Fig 1)	Selectivity (Fig 3)	Washout (Fig 4)	Wash in (Fig 5)
• **<P60** ◦**# data points (# animals)** ◦**mean age**	• 47 (16) • P33	• 20 (5) • P36	• 24 (11) • P36	• 74 (24) • P34
**>P60, # data points (# animals)**	24 (9)	10 (4)	11 (7)	39 (13)

Recordings were made using an Axopatch 1-D (Molecular Devices), filtered at 5 kHz and collected at 20 kHz using custom procedures written for NIDAQ and IGOR Pro (National Instruments and WaveMetrics, respectively). In control animals, neurons were selected from throughout the VTA. All experiments were completed in voltage clamp, V_holding_ = −60 mV. Input resistance and series resistance were measured once every 10 s with a 4 mV hyperpolarizing pulse. Any cells with more than 15% change in either measure during control periods between drug response measurements were eliminated from the study. Agonists were applied via pressure ejection (Smart Squirt, Automate, Inc.), 60 s per application, followed by 30 s of control aCSF, from a 250 μm tip placed within 350 μm of the recorded cell. Agonist solutions were loaded into the Smart Squirt at concentrations at least 10x saturating so that even with diffusion a saturating concentration of agonist would reach the recorded cell.

Agonists, antagonists, ATP, GTP, and all other chemicals were obtained from Sigma or Tocris Bioscience. KOR antagonists were provided by BlackThorn Therapeutics, Inc.

### *In vitro* pharmacology at rat KOR

Cellular antagonist effects of BTRX-335140 and BTRX-395750 (0.3 nM– 0.3 μM) were assessed in duplicate using in a rat recombinant CHO cell line using a cAMP-based time-resolved FRET assay (Eurofins Cerep, France). Results were calculated as a percent inhibition following application of the KOR agonist, (-)-U50,488 (3 nM).

### Data analysis

Each agonist response was calculated as the difference in the *I*_holding_ between the 2 min just prior to the agonist application and the 30 s around the peak response. Results are presented as mean ± SEM. Violin plots were constructed from the kernel density estimate of the data, where the bandwidth of the kernel was set to the range of the data in the plot divided by 10. Over 80% of concentration response experiments and wash-in measurements were made blind to antagonist identity. Concentration response data were fit to a 4-parameter log-logistic dose response model using the drc package in R, setting the upper asymptote to 100%, to estimate IC_50_, its variance, and lower asymptote (maximum blockade). Paired t-tests, two tailed, (vassarstats.net) were conducted to compare within cell baseline responses to MOR and DOR agonists with responses to these agonists in the presence of the KOR antagonists. A small number of DAMGO responses observed here were inward currents, consistent with prior observations [1]; in order to include these responses in the statistical evaluation of whether the KOR antagonists impacted the magnitude of the DAMGO responses, the paired t-tests were performed on the absolute values of the responses. Washout was statistically evaluated with a linear mixed effects model (JASP). Levene’s test for homogeneity of variance and Kruskal-Wallis rank sum test were performed in R to evaluate the wash in data. Statistical significance was set at *p* < 0.05. Data are available on OSF (DOI 10.17605/OSF.IO/AURZ7).

## Results

### Concentration responses for the KOR antagonists

**Fig 1:**
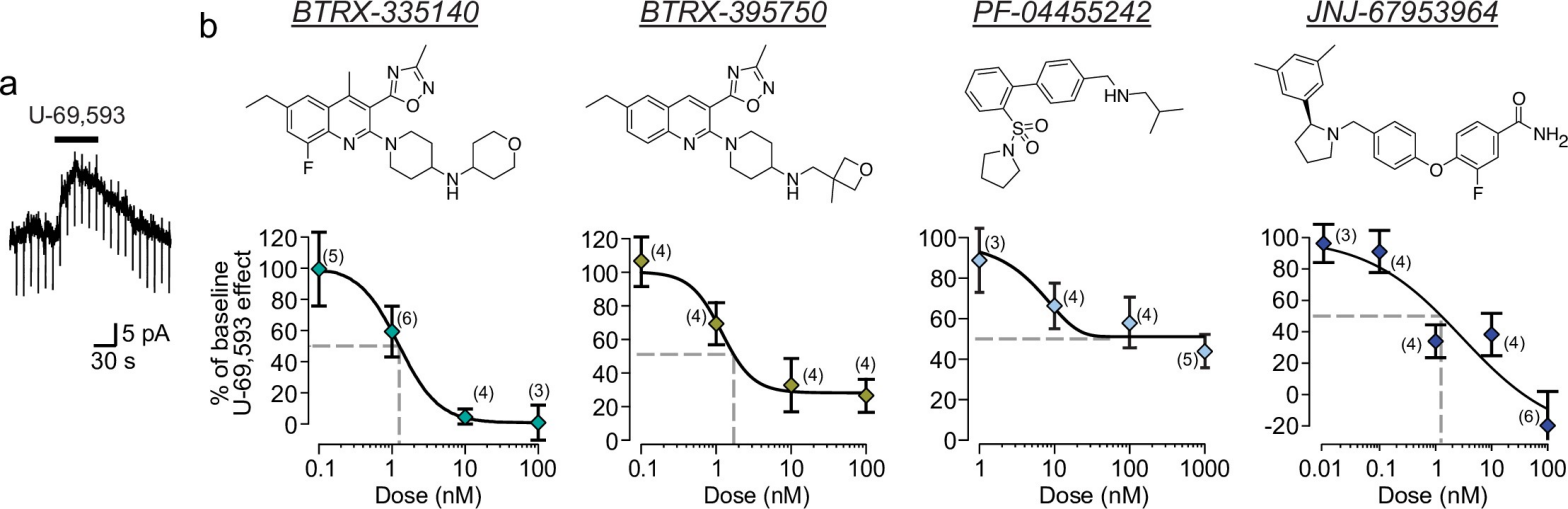
Concentration response relationships for blockade of U-69,593 induced K^+^ currents in VTA dopamine neurons by novel KOR antagonists. **a** Example voltage clamp recording of an outward current in a VTA neuron in response to pressure ejection of the KOR agonist U-69,593. **b** Top row, structures of the 4 KOR antagonists tested. Bottom row, concentration response curves for blockade of U-69,593 responses for four recently developed KOR antagonists. Number of replicates for each concentration tested is indicated in parentheses. Fits were generated with a 4-parameter log-logistic dose response model. Grey dashed lines indicate the concentration required to block 50% of the baseline U-69,593 responses. Data shown as mean ± SEM.

Responses of VTA neurons to pressure ejection application of a super-saturating concentration of the KOR agonist U-69,593 were measured in acute horizontal brain slices from rats using whole cell electrophysiology in voltage clamp configuration. KOR activation under these conditions activates K^+^ channels in many neurons, which in voltage clamp mode results in an outward (positive) current deflection (Fig 1A). Approximately half of VTA dopamine neurons are hyperpolarized by KOR activation [28], therefore each cell was tested for a U-69,593 response, and those that responded with an outward current were used to measure the efficacy of an antagonist to block the response to subsequent re-application of U-69,593. In control experiments of repeated U-69,593 testing without addition of antagonists, we found no evidence for desensitization of the U-69,593 response in this preparation: the second responses were 124 ± 7% the magnitude of the first responses (n = 9). For BTRX-335140, we measured an IC_50_ of 1.2 ± 0.9 nM (Fig 1B). The lower asymptote of the fit approached 1.3% of baseline U-69,593 response. Both 10 and 100 nM blocked the U-69,593 responses to less than 10% of the baseline response. This is quite similar to our measurements in a CHO-based heterologous system expressing rat KORs, where we found that BTRX-335140 had an IC_50_ of 3.2 nM for blocking inhibition of adenylyl cylcase by (-)-U50,488 (3 nM). For a structurally related compound in the same series, BTRX-395750, we measured an IC_50_ of 1.2 ± 1.3 nM and asymptoting at 28.0% of baseline U-69,593 response (Fig 1B), a greater potency than was measured in the heterologous system (IC_50_ = 48 nM).

Although PF-04455242 is reported to be a full antagonist in heterologous systems [36], we found that it only partially blocked the U-69,593 responses in the electrophysiology assay (Fig 1B). We observed a maximal blockade asymptoting towards 45% of the baseline U-69,593 responses by 100 nM PF-04455242. The concentration of PF-04455242 that produced half of the maximum effect for this antagonist is 6.7 ± 15.1 nM. These data indicate that PF-04455242 is a partial antagonist in this tissue.

We also studied the concentration response of JNJ-67953964, which yielded an IC_50_ of 3.0 ± 4.6 nM (Fig 1B). A surprising result in these experiments was that in the presence of 100 nM JNJ-67953964, a subset of neurons responded to U-69,593 with inward currents instead of outward currents, indicating an off target impact of JNJ-67953964 on KOR signaling. An inward current was also observed in 1 of 2 neurons tested for responses to U-69,593 in the presence of 1 μM JNJ-67953964 (-22.5% and 34.0% of baseline U-69,593 responses). These inward currents also drove the minimum asymptote to -27% of baseline U-69,593 responses.

**Fig 2:**
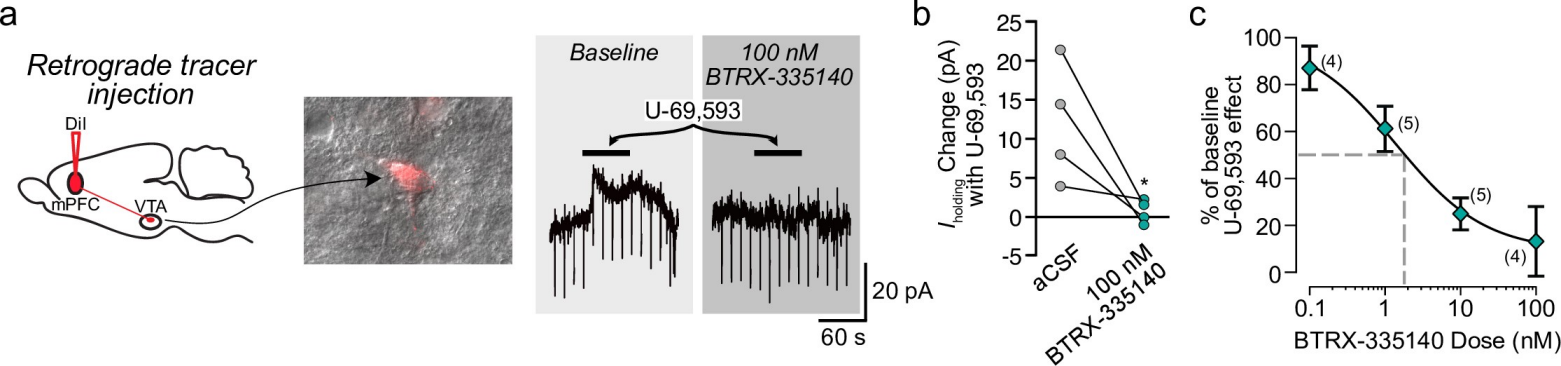
The KOR antagonist BTRX-335140 blocks U-69,593 responses in mPFC-projecting VTA dopamine neurons. **a** Example VTA neuron retrogradely labeled by stereotaxic injection of DiI into the mPFC (left). This neuron responded to U-69,593 application, and this outward current was completely blocked by the KOR antagonist BTRX-335140 (right). Across neurons (**b**), 100 nM BTRX-335140 significantly decreased the change in *I*_holding_ induced by U-69,593 (paired t-test, *p* = 0.03). The concentration response relationship for BTRX-335140 specifically in VTA neurons that project to the mPFC (**c**) is similar to that measured among unselected neurons (Fig 1B).

We have previously determined that KOR activation specifically inhibits VTA dopamine neurons that project to the mPFC but not to the NAc [37]. To investigate the effects of KOR antagonism on this specific circuit, we measured the concentration response of BTRX-335140 blockade of U-69,593 responses in VTA dopamine neurons that project to the mPFC. The fluorescent tracer DiI was injected into the mPFC 7–8 days prior to *ex vivo* recordings, and was detected in somata prior to patching (Fig 2A). Consistent with prior observations [37], outward currents were observed in response to U-69,593 in VTA neurons that project to the mPFC, and these responses were blocked by BTRX-335140 (Fig 2A and 2B). In these selected neurons, the IC_50_ of BTRX-335140 was 1.4 ± 1.1 nM, similar to the IC_50_ determined among non-projection-selected neurons (Fig 2C).

### Selectivity of BTRX-335140 compared to JNJ-67953964 for KORs

**Fig 3:**
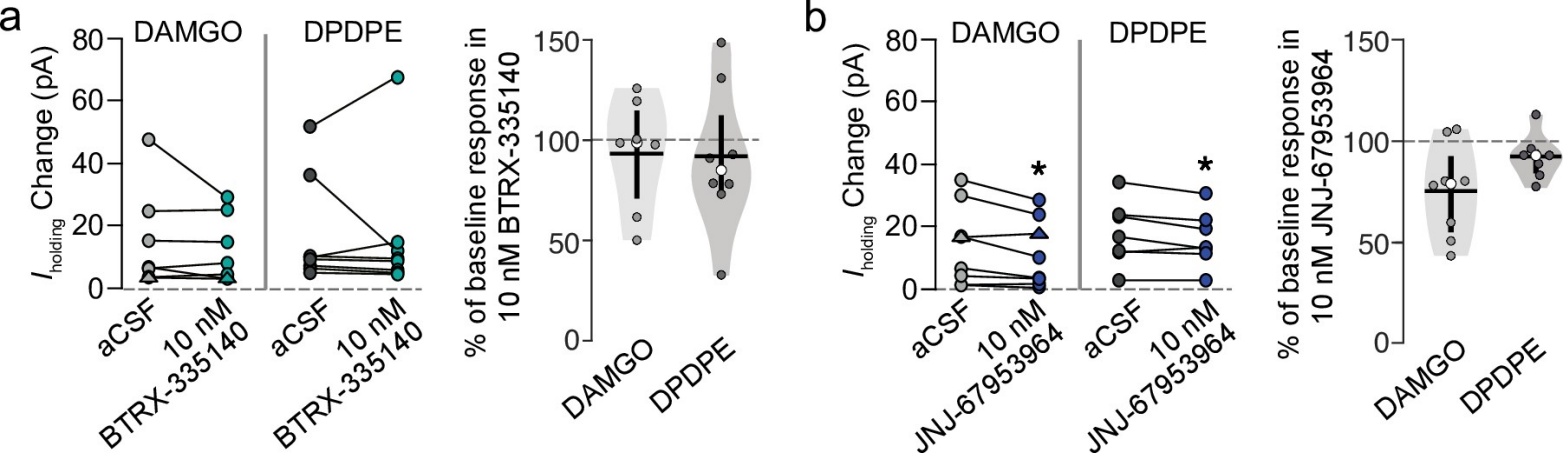
JNJ-67953964 is less selective for KOR over other opioid receptors compared to BTRX-335140 in VTA neurons. BTRX-335140 (**a**) and JNJ-67953964 (**b**) were tested for off target blockade of MOR or DOR with the agonists DAMGO and DPDPE, respectively. Neurons that responded with a change in holding current in response to DAMGO or DPDPE were re-tested with the agonist in the presence of 10 nM of the antagonist (left panels of **a** and **b**). The small number of neurons that responded to DAMGO with an inward current are illustrated here with triangles after taking the absolute value of the change in *I*_holding_. All data were also transformed to show the percent of the baseline response (right panel of **a** and **b**). Median percent of baseline shown in white circles, mean shown in horizontal black bars, twenty fifth and 75^th^ percentiles indicated by vertical black bars. *p < 0.05.

To evaluate the selectivity of BTRX-335140 and JNJ-67953964 for KORs over MORs and DORs, we tested their ability to block selective agonist-induced responses at these two receptors in VTA neurons. Super-saturating concentrations of the MOR selective agonist DAMGO (10 μM) or the DOR selective agonist DPDPE (10 μM) were pressure ejected onto VTA neurons in the same manner as U-69,593. We have previously shown that DAMGO and DPDPE responses in VTA neurons in acute brain slices do not desensitize [1, 2]. In responsive neurons, the agonist was re-applied after 10 nM of either KOR antagonist had been bath applied for at least 4 min. This concentration of BTRX-335140, which acted as a full antagonist to block U-69,593 responses, did not affect responses to DAMGO (n = 7, paired t-test *t* = +1.06, *df* = 6, *p* = 0.16) or DPDPE (n = 8, paired t-test *t* = +0.25, *df* = 7, *p* = 0.4; Fig 3A). By contrast, a 10 nM concentration of JNJ-67953964, which greatly diminished U-69,593 responses, consistently decreased responses to DAMGO (n = 8, paired t-test *t* = +2.54, *df* = 7, *p* = 0.019) and to a smaller extent inhibited responses to DPDPE (n = 7, paired t-test *t* = +2.25, *df* = 6, *p* = 0.033; Fig 3B).

### Wash out of test antagonists

One major shortcoming of norBNI, the KOR antagonist used most broadly in preclinical studies, is the persistent effects of a single administration [20, 24]. A short acting, selective antagonist is not only useful for clinical development, but also for experimental designs that require ligands to reverse rapidly enough for repeated administrations to have discernible effects. Here we measured whether responses to U-69,593 recovered after 10 or 20 min of washout of the novel antagonists. Concentrations of antagonists were chosen for their maximal blockade of U-69,593 effects in the concentration response experiments.

**Fig 4:**
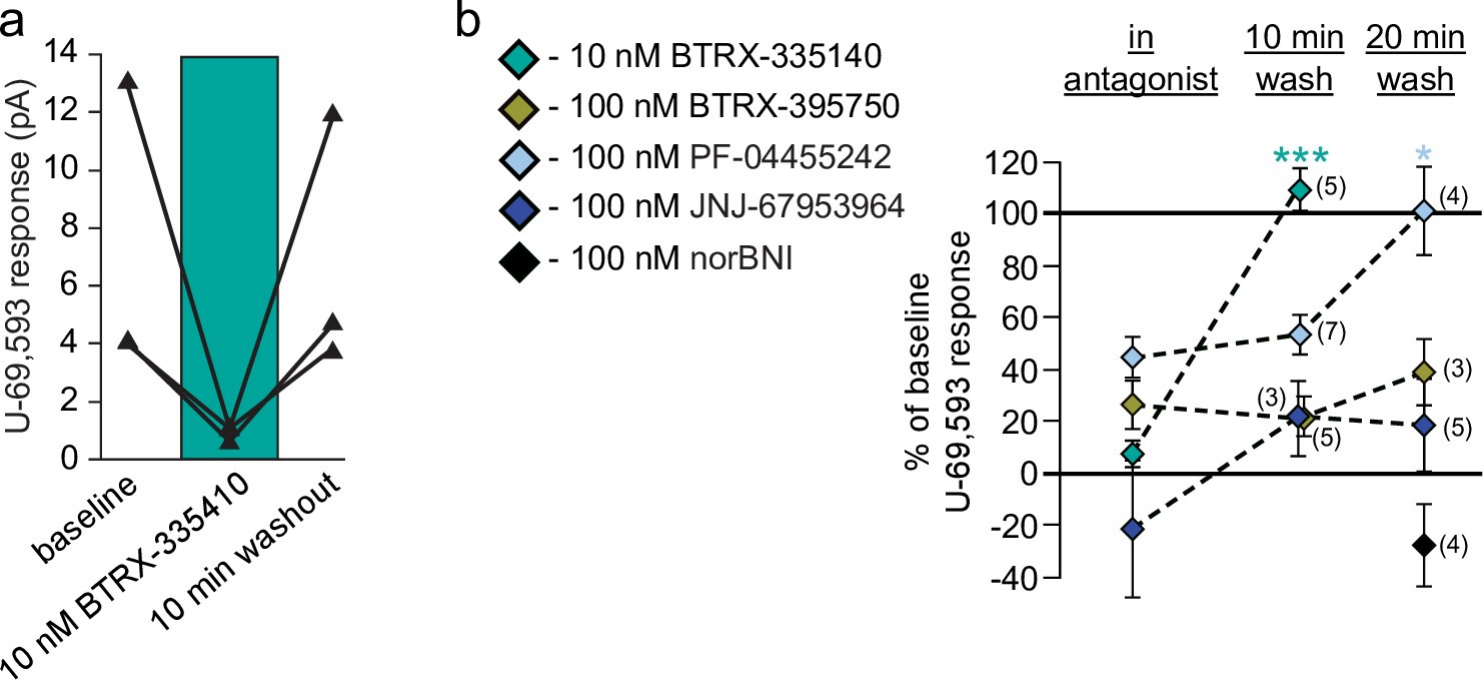
Washout of some novel KOR antagonists can be detected in VTA dopamine neurons. **a** Data from 3 different example neurons showing that 10 nM BTRX-335140 blocked U-69,593 responses, and this blockade reversed following 10 min of antagonist washout. **b** Summary across all neurons and antagonists showing that only BTRX-335140 reverses within 10 min. The partial antagonism of PF-04455242 reversed at 20 min. Number of observations indicated in parentheses. “In antagonist” data are same as in Fig 1. Mean ± SEM shown in panel b. *p < 0.05, ***p < 0.001.

In each experiment, a baseline U-69,593 response was measured, then the antagonist was applied to the slice for at least 5 min. This interval and the antagonist concentration selected were conditions sufficient to completely block U-69,593 responses, as observed in concentration response experiments shown above. U-69,593 responses were then probed 10 and/or 20 minutes after antagonist washout commenced, depending on the duration of the stability the whole cell recording. As expected, application of a concentration of norBNI (100 nM) that we have previously used to either fully block [28] or reverse [38] U-69,593 effects in VTA neurons showed no reversal at 20 min of washout (Fig 4B). On the other hand, a 10 nM concentration of BTRX-335140, sufficient to completely block U-69,593 actions (Figs 1B and 4A), showed complete washout within 10 min (linear mixed model: *df* = 1,7, *F* = 92.3, *p* < 0.001). Interestingly, BTRX-395750, which is structurally closely related to BTRX- 335140 (Fig 1B), did not show clear reversal with up to 20 min washout (Fig 4B; linear mixed model: *df* = 2,2.91, *F* = 1.3, *p* = 0.4). PF-04455242 persisted through 10 min, but did permit recovery of the U-69,593 effect at 20 min (Fig 4B; linear mixed model: *df* = 2,12, *F* = 4.9, *p* = 0.03). JNJ-67953964 on average showed some apparent washout, but only to the extent that the inward currents produced by U-69,593 in the presence of JNJ-67953964 in some neurons (Fig 1B) were not observed at either washout timepoint (Fig 4B; linear mixed model: *df* = 2,2, *F* = 7.6, *p* = 0.1).

### Wash in effects of test antagonists

**Fig 5:**
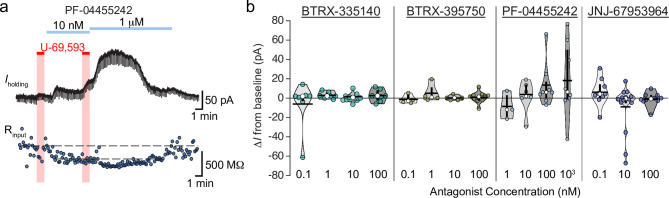
Among novel KOR antagonists, PF-04455242 stands out as activating a current. **a** Current (top) and input resistance (bottom) of example voltage clamp recording. This neuron responded to U-69,593 with a small outward current and decrease in input resistance. This neuron responded to PF-04455242 with a larger outward current and concurrent decrease in input resistance in a concentration dependent manner. **b** Summary of all changes in holding current in response to bath application to a range of concentrations of each KOR antagonist. No significant difference between the means of the groups was detected. Median percent of baseline shown in white circles, mean shown in horizontal black bars, twenty fifth and 75^th^ percentiles indicated by vertical black bars.

In VTA slices, we have not observed effects of norBNI that would suggest either constitutive activity at the KOR or endogenous dynorphin release in midbrain tissue from naïve animals [28, 38]. Therefore, neutral antagonists at the KOR would not be expected to drive any change in *I*_holding_ in voltage clamp recordings. Here we measured the change in *I*_holding_ following bath application of each antagonist at multiple concentrations. We detected a borderline unequal variance across antagonist-concentration groups (Levene’s test for homogeneity of variance, *F* = 1.75, *p* = 0.05), therefore to be conservative we used a non-parametric test to evaluate differences between groups; there was no significant difference between the means of these groups (Kruskal-Wallis rank sum test χ^2^ = 18.4, *p* = 0.24). This raises the possibility that a small subpopulation of neurons did respond to wash in of an antagonist with a shift in *I*_holding_, sufficient to increase the variance but not observed with a high enough frequency to drive a significant difference in means. BTRX-335140 and BTRX-395750 wash in measures all showed small variances across the concentrations tested, with means very close to zero (Fig 5B). Interestingly, PF-04455242 did induce a shift in *I*_holding_ in a subset of neurons (at 100 nM, 2 of 8 neurons, at 1 μM, 4 of 8 neurons; Fig 5A and 5B). This change in *I*_holding_, in all cases but one outward in nature, was accompanied by a decrease in input resistance, consistent with a channel opening (Fig 5A). Given the intracellular and extracellular solutions used here, and that the neurons were clamped at V_m_ = -60 mV, the outward currents are most likely K^+^ mediated, such as through a G protein coupled inwardly rectifying K^+^ channel (GIRK). GIRK is the typical postsynaptic coupled ion channel for opioid receptors, however, since every neuron tested in these wash in experiments responded to the KOR agonist U-69,593, yet only a subset of them show this response to PF-04455242, it is unlikely that these currents are due to activation of KORs. We also observed a possible inward current in a small subpopulation of neurons specifically at the 10 nM concentration of JNJ-67953964 (3 of 13; Fig 5B).

## Discussion

Here we tested the potency, selectivity, and reversibility of several recently developed KOR antagonists using electrophysiological measurements in acute rat brain slices. This preparation has the advantage of measuring ligand actions at receptors expressed endogenously in the mammalian brain. We selected the VTA dopaminergic neurons for profiling these molecules because this is a brain region where KOR agonists produce potent aversive actions, and because many other GPCRs are also expressed in VTA neurons, increasing the likelihood that any off-target effects of these ligands could be detected. In fact, this approach did reveal some unpredicted properties of some of these putative KOR antagonists. Further, it enabled us to test for reversal during washout, neural modulation with wash in, and off target blockade of endogenously expressed MORs or DORs. BTRX-335140 in particular was potent (1.2 nM IC_50_), showed rapid reversal of KOR antagonism in washout, and lacked MOR or DOR antagonist effects at the concentrations tested. In preclinical studies [26], BTRX-335140 showed oral efficacy in target engagement measures and is currently in clinical development.

BTRX-395750 also exhibited <10 nM potency at the KOR in this preparation, however it showed no significant washout of KOR blockade up to 20 min. This is interesting given that this molecule is within the same chemical series as BTRX-335140 and they are structurally closely related. Understanding how the specific differences in the structures affect the molecules’ orientations in the KOR binding pocket may inform what receptor-ligand interactions contribute to receptor residence times for the KOR. This is particularly interesting given the anomalously long duration of action of not only norBNI, but also the nonmorphinan KOR antagonist JDTic [21].

PF-04455242 showed some unexpected results in the characterization studies performed here compared to previously described pharmacological properties [27, 36]. First, we found it to only have partial antagonist action, with maximal blockade of the U-69,593 response plateauing at approximately 50%. We also found that PF-04455242 appears to activate a K^+^ conductance on a subset of the recorded cells. It is unlikely that these responses were due to activation of KOR, since all of the neurons tested with the compound initially responded to U-69,593. Consistent with these responses not being due to KOR activation, in HEK293 cells expressing KOR, PF-04455242 did not generate *extracellular signal-regulated kinase* (ERK) phosphorylation and induced minimal *c-Jun N terminal kinase* (JNK) phosphorylation compared to other KOR antagonists [21]. This compound was reported to have only moderate binding selectivity for KOR over MOR in radioligand displacement studies in rat brain tissue [36]. Together, these observations indicate PF-04455242 is quite different from a neutral KOR selective antagonist.

JNJ-67953964 effects have been explored in a variety of preclinical and human studies. It is brain-penetrant and well tolerated in humans [39–41], including in people in early abstinence from cocaine dependence [42]. In heterologous systems this compound acts as a neutral antagonist at KOR and at higher concentrations also blocks MOR [23]. However in our acute brain slice preparation, we detected an unexpected effect at the KOR in a subset of neurons, wherein JNJ-67953964 at 100 nM switched the expected outward current driven by KOR activation to an inward current. This change in signaling appeared to wash out acutely within 10 min. It is unclear if this change in signaling is due to a JNJ-67953964 interaction with KOR, or through an action at another receptor. Since this switch was not uniform across all neurons tested, a direct action at KOR seems insufficient to explain how this effect would only be observed in a subset of neurons. While this effect is unique compared to the other antagonists investigated here, since it was only observed at relatively high concentrations, it may not be a major concern following systemic drug administration. On the other hand, it may be a confound in animal studies where JNJ-67953964 is centrally administered e.g. [43]. In our selectivity experiments, we also saw modest blockade of our DAMGO and DPDPE induced effects at just 10 nM JNJ-67953964. While some MOR antagonism has been reported for this compound previously [23, 40], we were surprised to detect it at this concentration, which did not achieve full KOR blockade in this preparation. Wash in of JNJ-67953964 seemed to generate an inward current in a subset of neurons, and surprisingly this was observed more often at 10 nM than at 100 nM. We previously found that a small subset of VTA neurons respond to nociceptin/orphanin FQ with an inward current only at low concentrations, peaking at 10 nM [44], and CRF also increases VTA glutamatergic EPSCs at lower concentrations and inhibits them at higher concentrations [45], making such a pattern not entirely unprecedented. Together, these observations indicate potentially important, unanticipated properties of JNJ-67953964.

Here we characterized the pharmacological properties of putative reversible, KOR selective antagonists using whole cell recordings in acute brain slices. Our results provide novel and in some cases surprising findings on how these compounds work in individual neurons that endogenously express KORs. In particular, this approach enabled us to (1) better identify the activity of these compounds at the KOR and (2) identify off-target effects of these compounds that were not previously understood. Not only does this work reveal the functional differences between these compounds that inform their use in both preclinical studies and clinical development, this study provides direct evidence that electrophysiology in the acute brain slice preparation enables detection of molecular properties of novel compounds that are not easily detected in conventional heterologous receptor expression systems used for drug screening.
